# ANG II and Aldosterone Acting Centrally Participate in the Enhanced Sodium Intake in Water-Deprived Renovascular Hypertensive Rats

**DOI:** 10.3389/fphar.2021.679985

**Published:** 2021-05-25

**Authors:** Gabriela Maria Lucera, José Vanderlei Menani, Eduardo Colombari, Débora Simões Almeida Colombari

**Affiliations:** Department of Physiology and Pathology, School of Dentistry, Sao Paulo State University, Araraquara, Brazil

**Keywords:** 2K1C, aldosterone, brain stem, AT1 receptors, angiotensin II, lamina terminalis, hypertension

## Abstract

Renovascular hypertension is a type of secondary hypertension caused by renal artery stenosis, leading to an increase in the renin–angiotensin–aldosterone system (RAAS). Two-kidney, 1-clip (2K1C) is a model of renovascular hypertension in which rats have an increased sodium intake induced by water deprivation (WD), a common situation found in the nature. In addition, a high-sodium diet in 2K1C rats induces glomerular lesion. Therefore, the purpose of this study was to investigate whether angiotensin II (ANG II) and/or aldosterone participates in the increased sodium intake in 2K1C rats under WD. In addition, we also verified if central AT1 and mineralocorticoid receptor blockade would change the high levels of arterial pressure in water-replete (WR) and WD 2K1C rats, because blood pressure changes can facilitate or inhibit water and sodium intake. Finally, possible central areas activated during WD or WD followed by partial rehydration (PR) in 2K1C rats were also investigated. Male Holtzman rats (150–180 g) received a silver clip around the left renal artery to induce renovascular hypertension. Six weeks after renal surgery, a stainless-steel cannula was implanted in the lateral ventricle, followed by 5–7 days of recovery before starting tests. Losartan (AT1 receptor antagonist) injected intracerebroventricularly attenuated water intake during the thirst test. Either icv losartan or RU28318 (mineralocorticoid receptor antagonist) reduced 0.3 M NaCl intake, whereas the combination of losartan and RU28318 icv totally blocked 0.3 M NaCl intake induced by WD in 2K1C rats. Losartan and RU28318 icv did not change hypertension levels of normohydrated 2K1C rats, but reduced the increase in mean arterial pressure (MAP) produced by WD. c-Fos expression increased in the lamina terminalis and in the NTS in WD condition, and increased even more after WD-PR. These results suggest the participation of ANG II and aldosterone acting centrally in the enhanced sodium intake in WD 2K1C rats, and not in the maintenance of hypertension in satiated and fluid-replete 2K1C rats.

## Introduction

Renovascular hypertension is a type of secondary hypertension caused mainly by atherosclerotic renal artery stenosis, followed by fibromuscular disease, arteritis, thrombosis, arterial dissection, and stenosis in a transplanted kidney [reviewed in (Derkx and Schalekamp, 1994; Textor, 2017; Herrmann and Textor, 2018)], and account for approximately 5–6% of high blood pressure cases in the elderly (Hansen et al., 2002). The increase in renin–angiotensin–aldosterone system (RAAS) activity has a pivotal role in the development of renovascular hypertension (Leenen et al., 1975; Lincevicius et al., 2015; Textor, 2017; Roncari et al., 2018). In fact, the central or peripheral administration of AT1 receptor or aldosterone receptor antagonist was efficient in decreasing the high blood pressure levels of renovascular hypertension similar to that in 2-kidney-1clip (2K1C) rats (Wilcox et al., 1996; de Oliveira-Sales et al., 2010; Lincevicius et al., 2015; Boshra and Abbas, 2017). In addition to hypertension, 2K1C rats display an increased daily sodium intake (Mohring et al., 1975; Roncari et al., 2018). It should be noted that sodium intake is one of the risk factors for kidney injury in 2K1C rats (Olson et al., 1986; Liu et al., 1993; Menendez-Castro et al., 2020).

Water deprivation (WD), which causes dehydration of the intra- and extracellular compartments, is a common disturbance observed in wildlife and in humans (McKinley and Johnson, 2004; Matsubayashi et al., 2007; Leshem et al., 2008; Leshem, 2009; Takei et al., 2012). Water conservation and sodium excretion by the kidneys, and thirst and sodium appetite, two motivated behavior states induced by WD, arise as responses to WD-induced hypovolemia and hyperosmolality (Weisinger et al., 1985; Sato et al., 1996; De Luca Jr et al., 2002; McKinley and Johnson, 2004; Bourque, 2008).

Thirst induced by WD occurs upon the activation of the osmoreceptors in the forebrain areas lacking blood–brain barrier, such as the subfornical organ (SFO) and the organum vasculosum of the lamina terminalis (OVLT), and also inside the blood–brain barrier, such as the median preoptic nucleus (MnPO); all of these three areas are part of the lamina terminalis in the forebrain (McKinley and Johnson, 2004; Bourque, 2008; Oldfield and McKinley, 2015). As a result of WD, hypovolemia triggers the activation of the RAAS and angiotensin II (ANG II) acting in AT1 receptors in the SFO, and OVLT also induces thirst (Barney et al., 1983; De Luca Jr et al., 2002; McKinley et al., 2003). Besides, as WD progressively advances during WD-induced hypovolemia, the high levels of ANG II acting in the forebrain, in the same areas cited above, also induce sodium intake (Weisinger et al., 1985; Sato et al., 1996; Johnson, 2007; Matsuda et al., 2017). In addition, aldosterone, another hormone of the RAAS, acting centrally also induces sodium appetite (Epstein, 1982; Sakai et al., 1986; Geerling et al., 2006a), and a synergistic effect of aldosterone and ANG II acting in the central nervous system has been demonstrated for sodium intake (Epstein, 1982; Sakai et al., 1986). The effects of aldosterone acting in the brain stem, particularly in the nucleus of the solitary tract (NTS), have been elegantly shown (Geerling et al., 2006a; Geerling et al., 2006b; Geerling and Loewy, 2009; Gasparini et al., 2018).

Although well known that 2K1C rats have an enhanced sodium intake induced by WD (Roncari et al., 2018), that sodium intake can worsen the glomerular injury caused by 2K1C hypertension (Olson et al., 1986; Liu et al., 1993; Menendez-Castro et al., 2020), and that these animals also have an increased RAAS (Leenen et al., 1975; Lincevicius et al., 2015; Textor, 2017; Roncari et al., 2018), it is not known if the high WD-induced sodium intake in these animals is due to the activation of ANG II and/or aldosterone receptors in the brain. Therefore, the purpose of the present study was to determine whether ANG II and/or aldosterone acting centrally participates in the increased WD-induced sodium intake in 2K1C rats. In addition, in 2K1C rats, we also aimed to verify if central AT1 and mineralocorticoid receptor blockade would change the high levels of arterial pressure in water-replete (WR) and WD 2K1C rats because blood pressure changes can facilitate or inhibit water and sodium intake (Evered, 1992; Thunhorst and Johnson, 1994). Finally, possible areas activated during WD or WD followed by partial rehydration (PR) were also investigated in 2K1C rats.

## Materials and Methods

### Experimental animals

Male Holtzman rats (initial weight of 150–180 g) were used. Animals were housed in individual stainless-steel cages with a free access to normal sodium diet (BioBase Rat Chow, Águas Frias, Brazil), water, and 0.3 M NaCl. Room temperature was maintained at 23 ± 2°C, humidity at 55 ± 10%, and on a 12:12 light–dark cycle as previously described (Roncari et al., 2018). Experimental protocols were approved by the Ethical Committee in Animal Use (CEUA) of the School of Dentistry-UNESP (Proc. CEUA 12/2018).

### Anesthesia and Euthanasia

Rats were anesthetized with ketamine [80 mg/kg of body weight (b. wt.)] combined with xylazine (7 mg/kg of b. wt.) for the surgeries. During the surgeries/procedures, the level of anesthesia was monitored by checking the eye blink reflex and a reaction to paw pinch, and was adjusted if necessary. Following the surgeries, animals received a prophylactic dose of penicillin (50,000 IU, intramuscularly) and a dose of the anti-inflammatory ketoprofen (1 mg/kg of b. wt, subcutaneously), as previously described (Barbosa et al., 2017). At the end of the experiments, rats were euthanized by placing them under deep anesthesia with sodium thiopental (100 mg/kg of b. wt, i. p.).

### Renovascular Hypertension

Rats were anesthetized as above, and the leſt renal artery was partially obstructed using a silver clip of 0.2 mm width as described before (Blanch et al., 2014; Barbosa et al., 2017). At the end of surgery, animals received postoperatory drugs as described above.

### Brain Surgery

As previously described (Roncari et al., 2018), rats were anesthetized, placed in a stereotaxic apparatus (Kopf, Tujunga, CA, United States), and the skull was leveled between the bregma and lambda. A stainless-steel 23-gauge cannula (12 × 0.6 mm) was implanted in direction to the lateral ventricle (LV) using the following coordinates: 0.1 mm rostral to the bregma, 1.4 mm lateral to the bregma, and 3.3 mm below the surface of the skull. The cannula was fixed to the cranium using dental acrylic resin and jeweler screws. Postsurgical treatment was administered as described above, and the rats could recover for one week before starting the experiments.

### Drug Injection into the Lateral Ventricle

Injections were made using 10 μL-Hamilton syringes connected by PE-10 polyethylene tubing to a needle that was introduced into the brain through the guide cannula, as previously described (Sato et al., 1996). At the time of testing, metal obturators were removed, and the injection cannula (2 mm longer than the guide cannula) was inserted into the guide cannula. Injection volumes into the LV ranged from 1 to 2 μl, as described below. The metal obturators were replaced after injections.

Losartan potassium, AT1 receptor antagonist (Sigma-Aldrich, St Louis, MO, United States), was administered at the dose of 66 µg/1 μl, as described previously (Sato et al., 1996), RU28318, mineralocorticoid receptor antagonist (Tocris Bioscience, Ellisville, MO, United States) was used at the dose of 100 ng/2 µl, as described previously (Kim et al., 1998), dissolved in 0.15 M NaCl (saline) and 1% ethanol in 0.15 M NaCl (vehicle).

### Water and 0.3 M NaCl Intake Tests

Daily, 0.3 M NaCl and water intake were recorded using 100 ml capacity polypropylene bottles. For salt appetite and thirst tests, the polypropylene bottles containing water and 0.3 M NaCl were removed from the cage, and burettes with 0.1 ml divisions fitted with metal drinking spouts were provided (Roncari et al., 2018).

### Arterial Pressure and Heart Rate Recordings

In the day before the arterial pressure recordings, animals were anesthetized as described above, and polyethylene tubing (PE-10 connected to a PE-50, Clay Adams, Parsippany, United States) was inserted into the abdominal aorta through the femoral artery, tunneled subcutaneously, and exposed on the back of the rat to allow access in conscious freely moving rats. To record pulsatile arterial pressure, mean arterial pressure (MAP), and heart rate (HR), the arterial catheter was connected to a Statham Gould (P23 Db) pressure transducer (Statham Gould, Cleveland, United States) coupled to a preamplifier (model ETH-200 Bridge Bio Amplifier, CBSciences Inc, Dover, United States) that was connected to a Powerlab computer data acquisition system (model Powerlab 16SP, ADInstruments, Castle Hill, Australia), as previously described (Roncari et al., 2018).

### Histology

At the end of the experiments, animals with LV cannulas received a single injection of 2% Evans blue solution (1 µl) into the LV (Roncari et al., 2018) and thereafter were deeply anesthetized with sodium thiopental as indicated above. The brains were removed and fixed in 10% formalin for at least 2 days. Following postfixation, the brains were frozen, cut in 60 µm sections, Giemsa-stained, and analyzed by light microscopy to confirm the central sites of the injections (Gasparini et al., 2018). For immunohistochemistry, the animals were deeply anesthetized with sodium thiopental. Then, they are transcardially perfused with 0.9% saline followed by 4% paraformaldehyde, as described previously (Barbosa et al., 2017; Speretta et al., 2019).

### Immunohistochemistry

Four sets of coronal sections (40 µm) of the brain stem and the forebrain were sectioned on a cryostat (Leica, CM 1850, Wetzlar, Hesse, Germany). Briefly, as described before (Barbosa et al., 2017; Speretta et al., 2019), one of the sets of the brain sections was preincubated for 15 min in a blocking solution composed of 10% (vol/vol) normal horse serum (NHS, Sigma, St Louis, MO, United States) and 0.3% (vol/vol) Triton X-100 (Sigma) in 0.1 M PBS, followed by rinses in PBS (3 × 10 min). Subsequently, sections were incubated with a rabbit anti–c-Fos antibody (1:1.500, Santa Cruz Biotechnology) in PBS containing 1% (vol/vol) NHS and 0.3% Triton-X-100 for 48 h at 4°C. Sections were rinsed in PBS between the steps. After this, a 1-h incubation period was maintained in donkey anti-rabbit Alexa Fluor 594 or 488 antibody (both 1:500; Life Technologies). Then, the sections were rinsed in PBS (3 × 5 min) and mounted onto slides in 0.5% gelatin and allowed to air-dry for 10–15 min before being covered and slipped using an anti-fade fluorescent mounting solution (Fluoromount, Sigma-Aldrich, St. Louis, MO, United States). The sections were examined in a fluorescence microscope (Leica DM5500 B, Wetzlar, Hessen, Germany) with the appropriate filters.

### Experimental protocols

All experiments were done 6–7 weeks after 2K1C, a phase where hypertension reached the plateau (Blanch et al., 2014; Oliveira-Sales et al., 2014; Barbosa et al., 2017). At the end of the sixth week, a guide cannula was implanted in the LV, and after 5–7 days the tests were started.

### Effects of AT1 or Mineralocorticoid, or the Combination of AT1 and Mineralocorticoid Antagonism on 0.3 M NaCl and Water Intake in Water-Deprived 2K1C Rats

Because WD induces thirst and sodium intake, we used the WD + PR protocol (WD-PR) to discriminate thirst from sodium appetite (Sato et al., 1996). Water was removed from the cage, and rats were maintained during 24 h with access to only food pellets. After this, water-deprived rats were allowed to drink only water until satiety (PR), and then animals had access to a hypertonic NaCl solution (0.3 M NaCl) for a salt appetite test. During the salt appetite test, water intake was also recorded. Rats had no access to food during PR and the sodium intake test.

In group 1, 2K1C rats were separated in WR (control group) or 24-h WD. Fifteen min before getting access to water (PR-period), food was removed from the cages, and animals received either losartan (66 µg/1 µl) or saline (0.15 M NaCl, 1 µl) into the LV. Cumulative water intake was recorded every 30 min for 2 h. At the end of PR, rats were immediately offered a burette containing 0.3 M NaCl (salt appetite test), and the cumulative intake of both water and 0.3 M NaCl was recorded for an additional 2-h period at every 30 min. At the end of the recording period, food was returned to the cages. Rats were tested on two different days with at least a 3-day interval. In each test, half of the WD and half of the WR rats received losartan, and the other half received saline into the LV. In the next test, rats received the same treatments into the LV in a counterbalanced design.

In group 2, 2K1C rats were separated in control WR or 24-h WD. Fifteen min before getting access to water (PR-period), all animals received either RU28318 (100 ng/2 µl) or vehicle (1% of ethanol in 0.15 M/2 µl) into the LV. The 0.3 M NaCl and water intake were recorded as in group 1. At the end of the recording period, food was returned to the cages. Rats were also tested on two different days with at least a 3-day interval, similar to the tests in group 1, except that RU28318 and vehicle were injected into the LV instead of losartan and saline.

In group 3, only WD rats were tested. Rats were also tested on two different days, with a 3-days interval. In the first test, after 24 h of WD, half of the group of WD 2K1C rats received RU28318 (100 ng/2 µl), and 10 min later, they received losartan (66 µg/1 µl), and the other half received vehicle (1% of ethanol in saline/2 µL), and 10 min later, they received saline (0.15 M NaCl, 1 µl) into the LV. After losartan or saline injection, the PR protocol was initiated, and 2 h later, 0.3 M NaCl burettes were also offered to the animals. 0.3 M NaCl and water intake were recorded as in groups 1 and 2. In the second test, rats received the same treatments into the LV in a counterbalanced design.

In all the three groups of animals, 3–4 days after the end of the last sodium appetite test, MAP and HR were recorded in conscious WR rats to verify the level of MAP and HR. Only results of intake tests from 2K1C rats with MAP higher than 150 mmHg were analyzed.

### Effects of the Combination of AT1 and Mineralocorticoid Antagonism on MAP and HR in WR and in Water-Deprived 2K1C Rats

In the fourth group of 2K1C, an arterial catheter was inserted into the femoral artery as described above. In the next day, MAP and HR were recorded in WR freely moving rats. After 20 min of MAP stabilization RU28318 (100 ng/2 µl) was injected into the LV, followed 10 min later by a second injection of losartan (66 ug/1 µl) and MAP and HR were recorded for an additional 4-h period.

In the fifth group of 2K1C rats, an arterial catheter was inserted into the femoral artery as described above. On the next day, MAP and HR were recorded in WR rats for a 4 h-period to get baseline MAP and HR during WR. At the end of the recording period, all rats were submitted to 24 -h WD, and MAP and HR were recorded again in 24-h WD freely moving rats. After 20 min of baseline recordings, RU28318 (100 ng/2 µl) was injected into the LV, followed 10 min later by a second injection of losartan (66 ug/1 µl), and MAP and HR were recorded for an additional 4-h period.

### Effects of Water Deprivation and Water Deprivation-Partial Rehydration on Fos Expression in the Forebrain and in the Brainstem

2K1C rats used for immunohistochemistry experiments were divided into three groups: WR, WD, and WD-PR. Immediately at the end of a WD or a WD-PR cycle, the animals were deeply anesthetized and perfused as described in the Methods. WR rats were also deeply anesthetized and perfused, and were used as controls. All rats were anesthetized and perfused in the morning period. The chosen areas in the lamina terminalis were MnPO/OVLT, since they are rich in AT1 receptors (Tsutsumi and Saavedra, 1991; Phillips and Sumners, 1998). Moreover, OVLT is devoid of blood–brain barrier, and MnPO is an integrative area in the lamina terminalis (McKinley et al., 2015; Oldfield and McKinley, 2015). The NTS, particularly the intermediate (iNTS) subnuclei, adjacent to the area postrema, has been shown to have mineralocorticoid receptors; therefore, it was also chosen (Geerling et al., 2006a; Geerling et al., 2006b). Fos-positive nuclei were counted by hand in MnPO/OVLT in one comparable section in all groups. For iNTS, we averaged the bilateral counting of three levels of the iNTS in all three groups. Neuroanatomical sites were identified according to Paxinos and Watson’s Atlas (Paxinos and Watson, 1986).

### Statistical Analysis

All data are expressed as means ± SEM. Two- or one-way analysis of variance (ANOVA) followed by the Student–Newman–Keuls *post hoc* test was used for comparisons. In all tests, differences were considered significant at *p* < 0.05 as described before (Ruchaya et al., 2016).

## Results

### Histological Analysis

**FIGURE 1:**
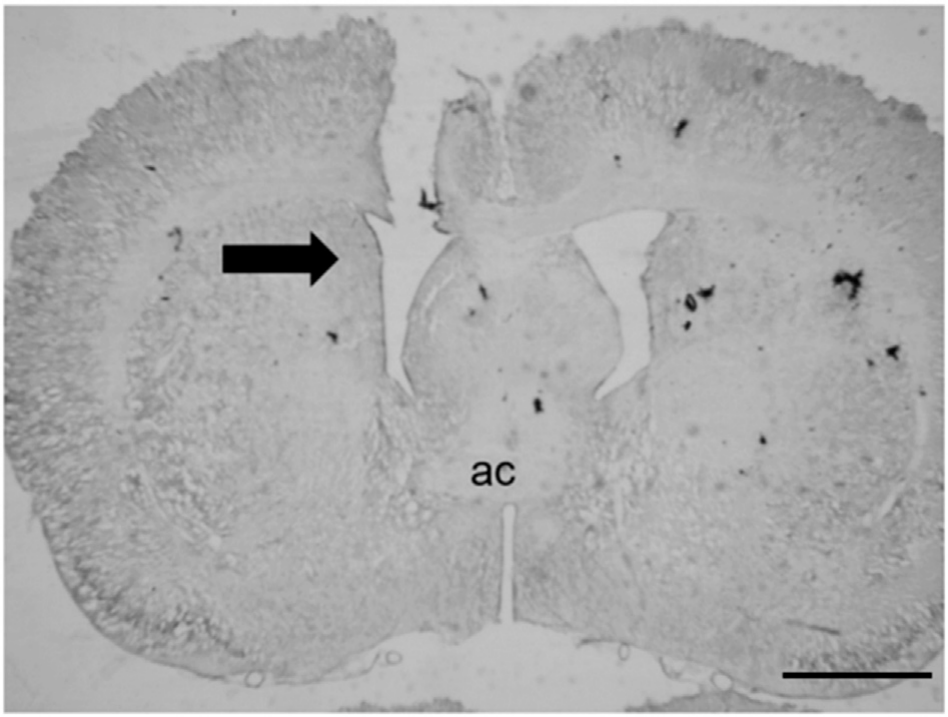
Representative photomicrograph of the tract of the cannula directed to the LV. The black arrow indicates the injection site; ac = anterior commissure (scale bar = 200 µm).

Figure 1 shows the tract of the cannula directed to the LV. The misplaced injections were either above, laterally, or caudally to the LV, and the data were analyzed separately.

### Effects of Central AT1 Receptor Antagonism on Water and 0.3 M NaCl Intake in Water-Deprived 2K1C Rats

**FIGURE 2:**
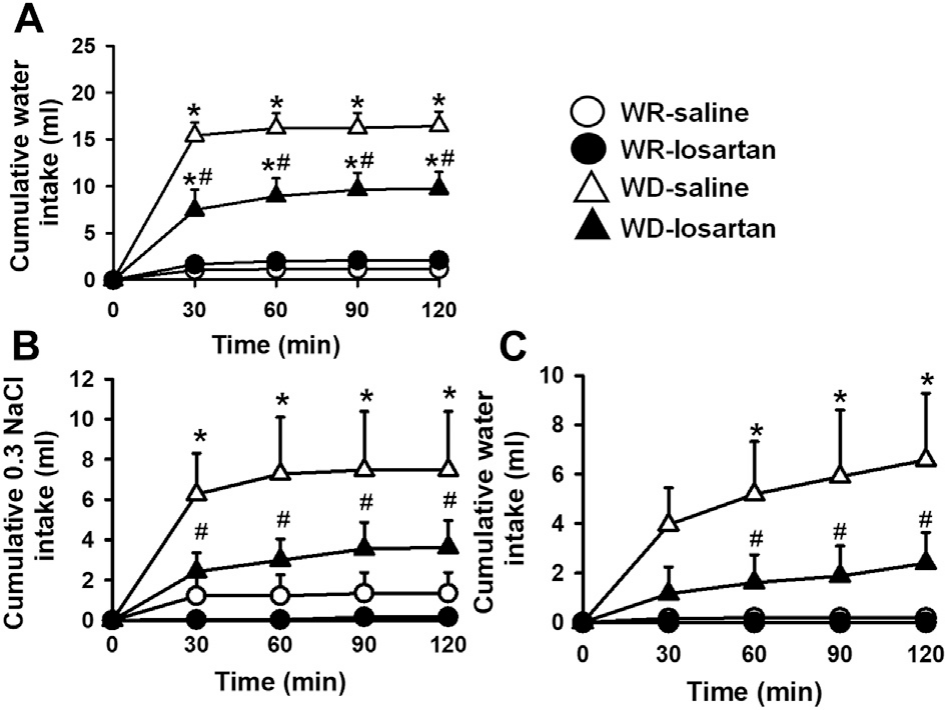
Cumulative water intake in the thirst test **(A)**, and cumulative 0.3 M NaCl **(B)** and water intake in the sodium appetite test **(C)** in water-replete (WR) or 24-h water-deprived (WD) 2K1C rats pretreated with losartan (66 μg/1 μl) or saline (1 μl) into the lateral ventricle. Mean arterial pressure (MAP) recording values are shown in text. Values are reported as the means ± SEM; *n* = 8; two-way analysis of variance (ANOVA) repeated measures, *p* < 0.05; * different from WR-saline; # different from WD-saline.

After 24 h of WD, 2K1C rats ingested significant amount of water compared to WR 2K1C rats (16.4 ± 1.5, vs. WR: 1.2 ± 0.4 ml/2 h; *p* < 0.05), a response reduced by the pretreatment with losartan (66 µg/1 µL) into the LV (9.8 ± 1.8 ml/2 h) [F (3, 21) = 26.17; *p* < 0.05], Figure 2. During the salt appetite test, WD 2K1C rats ingested significant amount of 0.3 M NaCl intake compared to WR 2K1C (7.5 ± 2.9, vs WR: 1.2 ± 0.4 ml/2 h), a response also reduced by the pretreatment with losartan into the LV (3.6 ± 1.3 ml/2 h) [F (3, 21) = 6.35; *p* < 0.05], Figure 2. In addition, water intake during the salt appetite test was also reduced by losartan into the LV in WD rats (2.4 ± 1.2, vs. saline: 6.6 ± 2.7 ml/2 h) [F (3, 21) = 5.11; *p* < 0.05], Figure 2. MAP and HR recorded 3–4 days after the last experiment were, respectively, 183 ± 3 mmHg and 402 ± 6 bpm.

### Effects of Central Mineralocorticoid Receptor Antagonism on Water and 0.3 M NaCl Intake in Water-Deprived 2K1C Rats

**FIGURE 3:**
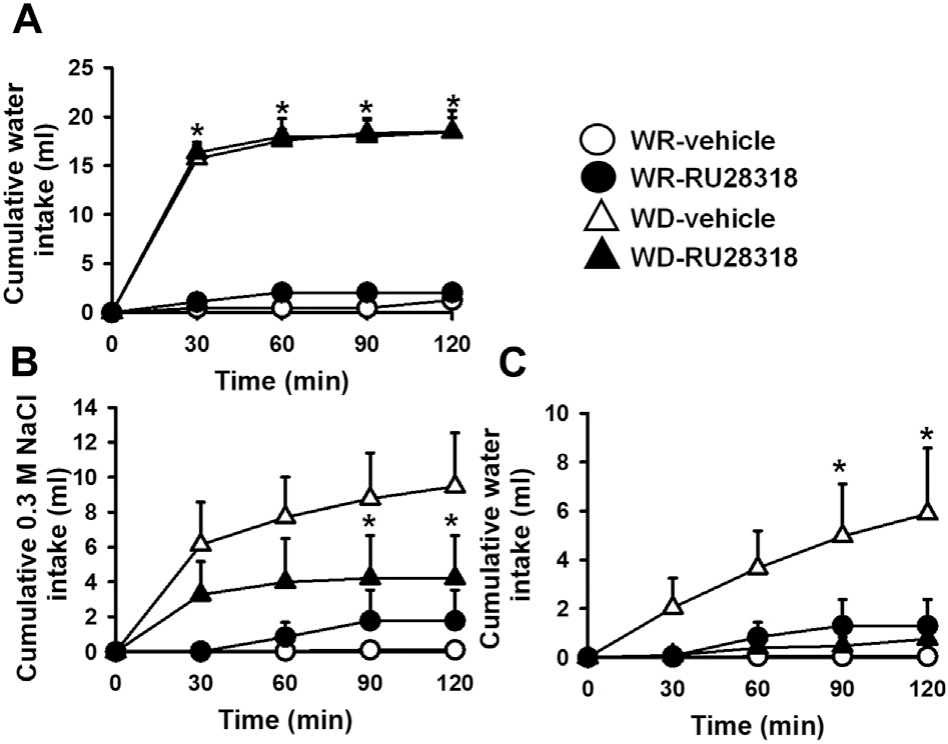
Cumulative water intake in the thirst test **(A)**, and cumulative 0.3 M NaCl **(B)** and water intake in the sodium appetite test **(C)** in WR or 24-h WD 2K1C rats pretreated with RU28318 (100 ng/2 μl) or vehicle (2 μl) into the lateral ventricle. MAP recording values are shown in text. Values are reported as means ± SEM; *n* = 7; two-way ANOVA repeated measures, *p* < 0.05; * different from WR-vehicle; # different from WD-vehicle.

After 24 h of WD, this group of 2K1C rats also ingested a significant amount of water compared to WR 2K1C (17.9 ± 1.3, vs. WR: 1.3 ± 0.3 ml/2 h) [F (3, 18) = 79.65; *p* < 0.05]. The pretreatment with RU28318 (100 ng/2 μl) into the LV did not modify WD-induced water intake (17.4 ± 2.1 ml/2 h; *p* > 0.05), Figure 3. During the salt appetite test, WD 2K1C rats presented a significant intake of 0.3 M NaCl compared to WR 2K1C rats (9.5 ± 3.1, vs. WR: 0.1 ± 0.1 ml/2 h) [F (3, 18) = 6.51; *p* < 0.05], Figure 3. Similar to losartan, the pretreatment with RU28318 into the LV reduced 0.3 M NaCl intake induced by WD (4.2 ± 2.5 ml/2 h) [F (3, 18) = 6.50; *p* < 0.05] and abolished water intake during the salt appetite test (0.8 ± 0.6, vs. vehicle: 5.9 ± 2.7 ml/2 h) [F (3, 18) = 4.66; *p* < 0.05], Figure 3. MAP and HR recorded 3–4 days after the last experiment were, respectively, 181 ± 3 mmHg and 382 ± 6 bpm.

### Effects of Combined Central AT1 and Mineralocorticoid Antagonism on Water and 0.3 M NaCl Intake in Water-Deprived 2K1C Rats

**FIGURE 4:**
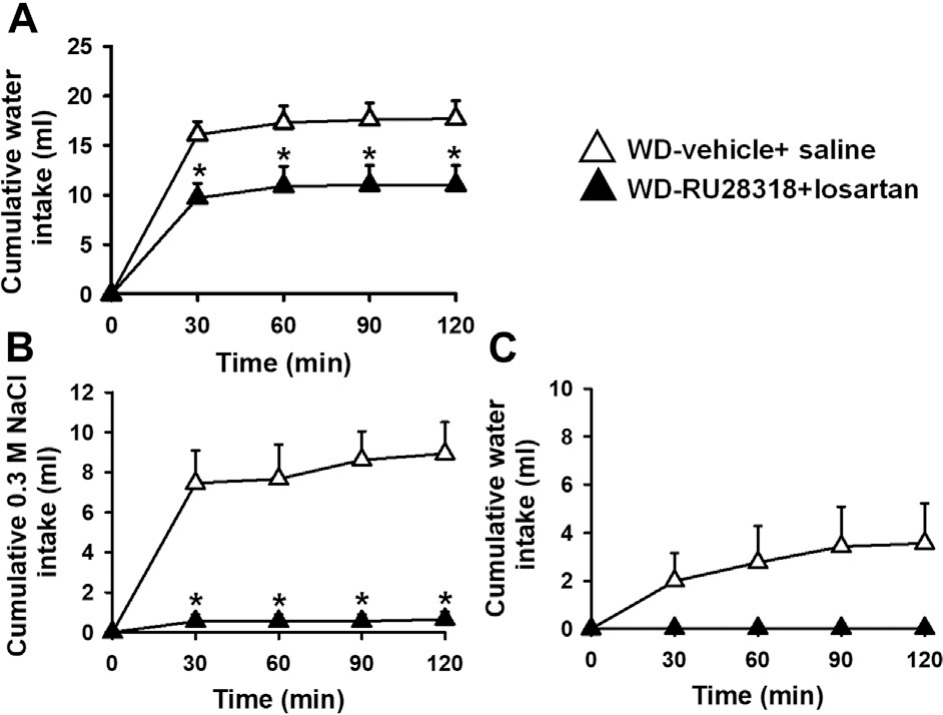
Cumulative water intake in the thirst test **(A)**, and cumulative 0.3 M NaCl **(B)** and water intake in the sodium appetite test **(C)** in 24-h WD 2K1C rats pretreated with RU28318 (100 ng/2 μl) + losartan (66 μg/1 μl) or vehicle + saline into the lateral ventricle. MAP recording values are shown in text. Values are reported as the means ± SEM; *n* = 8; two-way ANOVA repeated measures, *p* < 0.05; * different from WD-vehicle + saline.

The pretreatment with RU28318 (100 ng/2 μl) + losartan (66 µg/1 µL) icv reduced water intake induced by WD (11 ± 2, vs: vehicle + saline: 17.7 ± 1.8 ml/2 h) [F (1, 7) = 6.91; *p* < 0.05], Figure 4. Conversely, 0.3 M NaCl intake was totally blocked by the pretreatment with RU28318 + losartan icv (0.7 ± 0.4, vs. vehicle + saline: 8.9 ± 1.6 ml/2 h) [F (1, 7) = 27.14; *p* < 0.05], Figure 4. Water intake during the salt appetite was not different between RU28318 + losartan and vehicle + saline icv [F (1, 7) = 3.43; *p* > 0,05], Figure 4. In this group of rats, MAP and HR baseline levels were, respectively, 172 ± 3 mmHg and 395 ± 8 bpm.

### Effects of Combined Central AT1 and Mineralocorticoid Antagonism on MAP and HR of Water Replete and Water-Deprived 2K1C Rats

**FIGURE 5:**
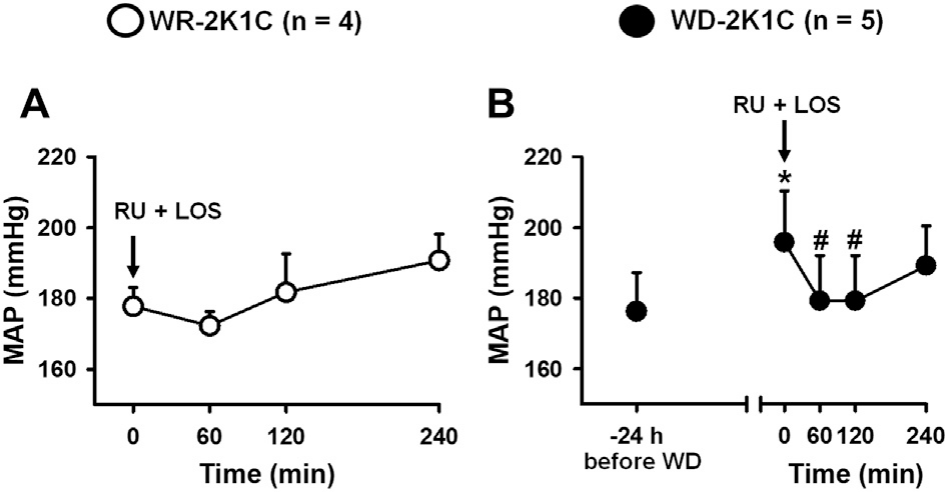
Change in MAP in **(A)** WR and **(B)** 24-h water-deprived rats after injection of RU28318 (100 ng/2 μl) + losartan (66 μg/1 μl) into the lateral ventricle. Values are reported as the means ± SEM; number of animals are within parentheses; one way ANOVA repeated measures, *p* < 0.05; * different from WR (before WD), # different from time 0 min.

In WR rats, the combination of RU28318 (100 ng/2 μl) + losartan (66 µg/1 µl) icv did not change baseline MAP (170 ± 6 before, vs. 174 ± 5 mmHg in the next 4 h after RU28318 + losartan) [F (5, 15) = 1.37; *p* > 0.05], Figure 5A, or HR (360 ± 2 before, vs. 387 ± 12 bpm in the next 4 h after RU28318 + losartan) [F (5, 15) = 1.03; *p* > 0.05]. MAP increased after 24 h of WD compared to baseline WR levels (169 ± 10, vs. WD: 188 ± 17 mmHg), Figure 5B. The combination of RU28318 + losartan icv abolished the increase of MAP in WD rats in the first 2 h to 179 ± 12 mmHg [F (4, 16) = 6.87; *p* < 0.05], Figure 5B. WD during 24 h did not change HR (WR: 401 ± 12, vs. WD: 408 ± 21 bpm; *p* > 0.05), and the combination of RU28318 + losartan also had no effect in HR (408 ± 21 before, vs. 381 ± 21 bpm after RU28318 + losartan) [F (4, 16) = 1.35; *p* > 0.05], Figure 5B.

### Misplaced Injections

Animals with injections outside the LV were pooled together as misplaced injections, and the 0.3 M NaCl and water intake were analyzed. Losartan or RU28318 alone or combined failed to reduce water intake or 0.3 M NaCl intake induced by WD (Table 1).

**TABLE 1:** Cumulative water intake during the thirst test and 0.3 M NaCl intake during the sodium appetite test in 2K1C submitted to 24-h water deprivation rats with misplaced injections of saline (0.15 M NaCl), losartan (66 µg/1 µl), vehicle (1% ethanol in 0.15 M NaCl), or RU28318 (100 ng/2 µl).

Treatment	Time (min)			
Water intake	30	60	90	120
Saline (*n* = 3)	19.7 ± 1	21.7 ± 1	21.9 ± 1	22.2 ± 1
Losartan (*n* = 3)	18.8 ± 1.3	21.6 ± 1.7	24.4 ± 2.1	24.7 ± 2.3
Vehicle (*n* = 4)	15.3 ± 0.8	17 ± 1.4	17.7 ± 1.7	18.3 ± 2
RU28318 (*n* = 4)	15 ± 1.1	16.8 ± 14	18.8 ± 1.7	19.2 ± 1.7
0.3 M NaCl intake	30	60	90	120
Saline (*n* = 3)	9.7 ± 2.8	13.5 ± 3.9	15.9 ± 4.6	17.5 ± 5.1
Losartan (*n* = 3)	8.0 ± 0.7	10.1 ± 1.3	13.4 ± 2.2	15.5 ± 2.5
Vehicle (*n* = 4)	12.9 ± 1.3	14.8 ± 1.1	15.8 ± 1.4	16.6 ± 1.5
RU28318 (*n* = 4)	6.9 ± 1.3	10 ± 1.5	12.2 ± 1.9	13 ± 2.2

Values are reported as the means ± SEM; *n* = number of animals.

### Expression of Fos in the MnPO/OVLT and iNTS in 2K1C Water Replete, Water-Deprived, and Water Deprived with Partial Rehydration.

**FIGURE 6:**
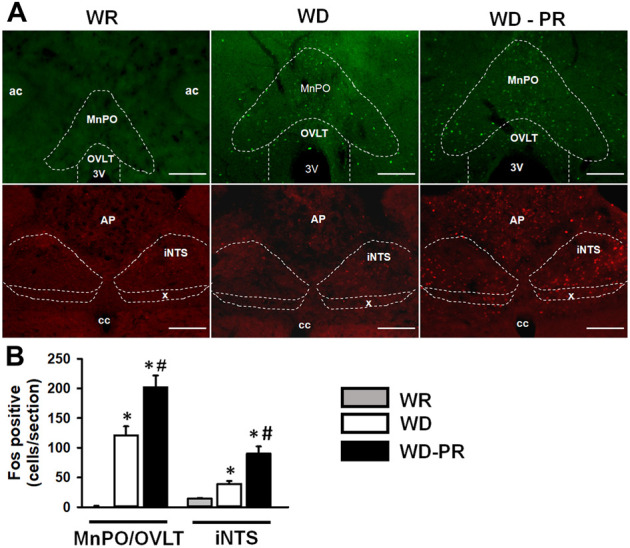
Representative photomicrographs **(A)** and graphic representations **(B)** of Fos immunoreactivity in the lamina terminalis (MnPO/OVLT) and in the intermediate nucleus of the solitary tract (iNTS) in WR, WD, and water-deprived partial rehydrated (WD-PR) 2K1C rats. In **(A)** ac = anterior commissure; MnPO = median preoptic nucleus; OVLT = organ vasculosum of the lamina terminalis; iNTS = intermediate nucleus of the solitary tract; AP = area postrema; x = dorsal motor nucleus of the vagus; cc = central canal; 3V = third ventricle; ×10 magnification; scale bar = 200 µm. In **(B)**, values are reported as means ± SEM; *n* = 3–6/group; one-way ANOVA; *p* < 0.001; * different from WR; # different from WD.

WD for of 24 h induced Fos expression in the lamina terminalis (MnPO/OVLT) compared with the WR rats (126 ± 18, vs. WR: 0.0 ± 0 cells/section), Figure 6. PR with water after 24 of WD (WD-PR) produced a further increment of Fos expression in the MnPO/OVLT compared to WD condition (201 ± 20 cells/section) [F (2, 8) = 35.35; *p* < 0.001], Figure 6. In the iNTS, WD also increased Fos expression when compared with WR rats (39 ± 5, vs. WR: 14 ± 1 cells/section), and WD-PR further increased Fos expression compared to WD condition (90 ± 11) [F (2, 21) = 24.95; *p* < 0.001] Figure 6.

## Discussion

The present results show that water intake induced by WD in 2K1C rats was reduced by icv losartan, not by icv RU2831, whereas 0.3 M NaCl intake was partially reduced either by icv losartan or RU28318, and totally abolished by the combined antagonism of central AT1 and mineralocorticoid receptors. The combined blockade of central AT1 and mineralocorticoid receptors did not change MAP levels in WR 2K1C, but suppressed the increase in MAP produced by WD in 2K1C rats. Finally, Fos expression in the MnPO/OVLT located in the forebrain, in the iNTS, and in the brain stem increased in WD 2K1C rats, and further increased after 2 h of rehydration.

Many studies have shown the importance of ANG II for water intake and sodium appetite (Sato et al., 1996; McKinley and Johnson, 2004; McKinley et al., 2015; Roncari et al., 2018). WD induces increased plasma osmolarity and renin levels that promote thirst and sodium appetite (Sato et al., 1996; De Luca Jr et al., 2002). Forebrain regions rich in ANG II AT1 receptor subtype such as SFO, MnPO, and OVLT participate in ANG II-induced thirst and sodium appetite, and are activated during WD (De Luca Jr et al., 2002). In the present study, water intake during the thirst test increased in WD 2K1C rats as previously demonstrated (Roncari et al., 2018). The present study extended the prior findings demonstrating that central AT1 receptor antagonism with losartan partially reduced thirst in WD 2K1C rats. Probably, water intake that remained after losartan icv was due to the activation of osmoreceptors as a consequence of the cell dehydration (McKinley, 1991).

Central AT1 receptor antagonism with losartan microinjection partially reduced sodium intake in WD 2K1C similar to the reduction produced by the antagonism of central mineralocorticoid receptor with RU28318 icv. In addition, the concurrent blockade of central AT1 and mineralocorticoid receptors completely blocked the sodium intake in WD 2K1C rats, suggesting that both mechanisms act simultaneously to induce sodium appetite in WD 2K1C rats. Epstein’s studies (Epstein, 1982; Sakai et al., 1986; Fluharty and Epstein, 2004) showed a synergism between ANG II and aldosterone to induce sodium intake in normotensive sodium depleted rats. In Sakai’s study (Sakai et al., 1986), RU28318 icv partially reduced sodium intake in normotensive sodium-depleted animals, and, when central blockade of mineralocorticoid receptors was combined with systemic captopril, to prevent endogenous ANG II formation during sodium depletion, sodium appetite was completely suppressed. Although aldosterone levels were not elevated during WD (Huang et al., 1996), 2K1C animals have greater baseline levels of circulating aldosterone due to the overactivation of RAAS (Leenen et al., 1975; Lincevicius et al., 2015). Thus, the present study demonstrated for the first time that the synergism between ANG II and aldosterone acting centrally is responsible for the expression of salt appetite in WD 2K1C rats. It should be noted that during the sodium appetite test of the WD-PR protocol used in the present study, rats also ingested water as a result of the hypertonicity caused by hypertonic NaCl intake.

Few brain sites contain specific aldosterone-sensitive cells, and the NTS, in the brain stem, is one of these sites (Geerling et al., 2006a; Geerling et al., 2006b). The neurons sensitive to aldosterone have, in addition to the expression of mineralocorticoid receptors, the enzyme 11-β-hydroxysteroid dehydrogenase type 2 (HSD2), which cleaves cortisol/corticosterone, present in high concentrations than aldosterone in the central nervous system, in a metabolite with few affinity for mineralocorticoid receptor, increasing the likelihood of aldosterone binding to mineralocorticoid receptor and having its effects (Geerling et al., 2006a; Geerling et al., 2006b).

The neurons that express mineralocorticoid receptors and HSD2 enzyme in the NTS are activated by aldosterone and project to forebrain regions, such as the bed nucleus of the stria terminalis (Geerling and Loewy, 2006). Circulating ANG II acts in the SFO and in the OVLT neurons that also project to bed nucleus of the stria terminalis (Sunn et al., 2003; Matsuda et al., 2017). In addition, a recent study from our laboratory demonstrated an interaction between aldosterone and ANG II acting in the brain stem and the forebrain, respectively, in the control of sodium appetite in normotensive animals (Gasparini et al., 2019a). A similar mechanism may act in WD 2K1C animals to induce sodium appetite, as suggested by the increase in Fos expression in the lamina terminalis and in the iNTS, particularly in WD-PR condition, when sodium appetite was tested.

Recent studies showed that blood–brain barrier is disrupted in 2K1C rats, particularly in the NTS, which may facilitate the action of circulating aldosterone on HSD2 neurons in this area (Fernandes et al., 2021). In addition, the NTS is located close to the area postrema, a circumventricular organ which is relatively accessible to circulating aldosterone (Geerling et al., 2006b; Shin et al., 2009). Indeed, systemic infusion of aldosterone increases the nuclear translocation of mineralocorticoid receptor in the NTS HSD2 neurons (Gasparini et al., 2019b). Although it is not possible to rule out the action of aldosterone in the forebrain, the volume of RU38238 injection in the LV was larger than that of losartan injection, which might allow the blockade of the mineralocorticoid receptors in the brain stem, by icv RU38238 reaching the fourth ventricle and the adjacent NTS. This hypothesis is further supported by Fos expression in the NTS, particularly in the iNTS, a local of HSD2-positive neurons (Geerling et al., 2006a; Geerling et al., 2006b; Shin et al., 2009).

Another issue analyzed was the possible influence of changes in blood pressure on the intake of water and/or sodium. It has been reported that an increase in blood pressure might inhibit the intake of water and sodium, while the reduction in blood pressure might facilitate these behaviors (Evered, 1992; Thunhorst and Johnson, 1994). In the present study, icv injection of RU28318 combined with losartan did not change MAP or HR in WR 2K1C rats, whereas the same treatment reduced the increase of MAP in WD 2K1C rats. Thus, there is no reason to expect that the combination of losartan and RU28318 icv might influence sodium intake in WD 2K1C rats. Interestingly, WD induced an increase in MAP, as shown in Figure 5, and the double antagonism restored blood pressure to the WR levels for about 120 min, suggesting that either ANG II or aldosterone or both are likely responsible for that further increase in MAP.

In conclusion, the present results suggest the participation of ANG II and aldosterone acting centrally in the enhanced sodium appetite of WD 2K1C rats, whereas only ANG II participates on the water intake in the thirst test. The lamina terminalis and iNTS activation during WD and WD-PR reinforce the role of both the forebrain and the hindbrain in the salt appetite test. In the experimental conditions tested, 2K1C rats with 0.3 M NaCl available to drink *ad libitum*, neither ANG II nor aldosterone acting centrally seems to be responsible for the high levels of MAP in 2K1C rats, but they were responsible for the further increase in MAP in WD 2K1C rats.

## Data Availability

The original contributions presented in the study are included in the article/Supplementary Material; further inquiries can be directed to the corresponding author.
