# Supplementary figures and images for: ANG II and Aldosterone Acting Centrally Participate in the Enhanced Sodium Intake in Water-Deprived Renovascular Hypertensive Rats
Source: Front Pharmacol. 2021 May 25;12:679985. doi: 10.3389/fphar.2021.679985 (PMC8186501; doi:10.3389/fphar.2021.679985)

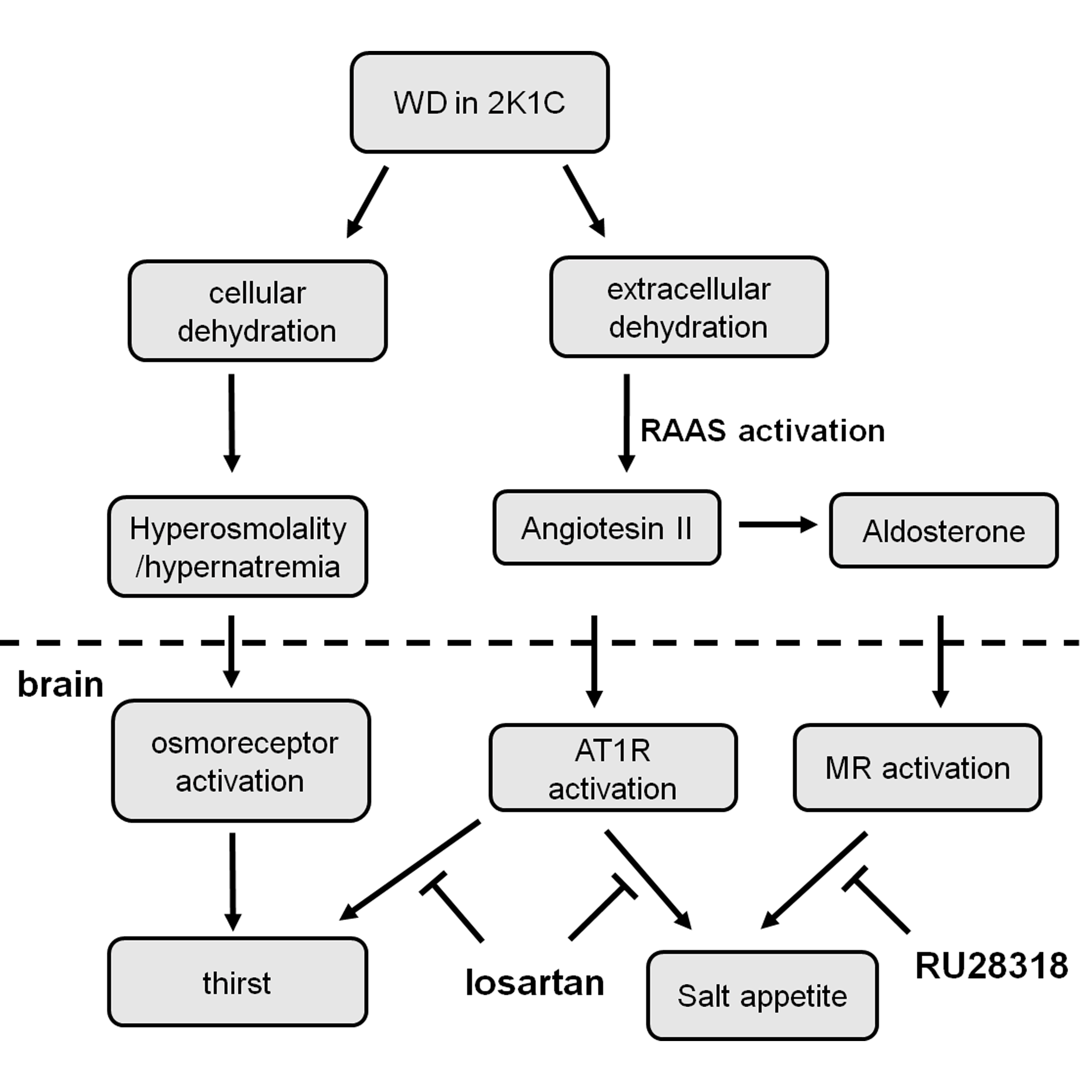

Supplement: Supplementary file 1 [file Image1.JPEG]
